# “It’s a waiting game” a qualitative study of the experience of carers of patients who require an alternate level of care

**DOI:** 10.1186/s12913-017-2272-6

**Published:** 2017-05-02

**Authors:** Kerry Kuluski, Jennifer Im, Mary McGeown

**Affiliations:** 1 Sinai Health System, Lunenfeld Tanenbaum Research Institute (Bridgepoint Hospital Site), 1 Bridgepoint Drive, M4M 2B5 Toronto, ON Canada; 2 Institute of Health Policy, Management and Evaluation, Dalla Lana School of Public Health, University of Toronto, Toronto, ON Canada; 3 Centre for Education and Research on Aging and Health, Lakehead University, Thunder Bay, ON Canada

**Keywords:** Hospital discharge, Alternate level of care, Carer, Caregiver, Care experience, Long-term care, Qualitative

## Abstract

**Background:**

Delayed hospital discharge (also known as Alternate Level of Care or ALC) is a global health care quality issue with negative implications for people (e.g., functional decline) and the health care system (e.g., costly interruptions in hospital flow and procedures). ALC disproportionately impacts people with cognitive impairment, and insight into the needs and experiences of this specific sub population and their carers is lacking. The purpose of this study was to understand the hospital experience of carers (e.g., family members) of patients with ALC and cognitive impairment who were waiting for long-term care from the hospital.

**Methods:**

This is a qualitative descriptive study entailing 12 semi-structured interviews with 15 carers of patients with ALC from three hospitals in Northwestern Ontario. Interviews were conducted between October 2015 and February 2016. Two reviewers thematically analyzed the interview data.

**Results:**

Five core themes were identified from the interview data: patient over person, uncertain and confusing process, inconsistent quality in care delivery, carers addressing gaps in the system, and personalization of long-term care.

**Conclusions:**

Waiting for long-term care from the hospital is a stressful and uncertain time for family carers. ALC is an ‘in-between’ phase when patients and carers may be at their most vulnerable yet receive the least care from the formal care system. Carers provide critical insight into the needs and behaviors of patients as well as processes that need to be improved to enhance their experience. Such insights will help health systems internationally as they grapple with the issue of ALC whilst trying to optimize engagement with patients and their families.

**Electronic supplementary material:**

The online version of this article (doi:10.1186/s12913-017-2272-6) contains supplementary material, which is available to authorized users.

## Background

Healthcare quality improvement sits high on the agenda across industrialized countries and is fueled by aging populations and increasing care needs. A high quality health system is defined as one that is safe, effective, patient centered, timely, efficient and equitable [1].

Poor quality outcomes can manifest in many different ways including lack of access to needed services, being on lengthy waiting lists, or being in a care setting that does not align with level of need. We see this latter problem through delayed hospital discharges, known in Canada as Alternate Level of Care (ALC). An ALC designation is given when a person has been medically cleared for discharge but remains in hospital due to lack of appropriate alternatives (e.g., homecare, assisted living or a long-term care (LTC) bed) [2]. ALC is a care quality issue that places people at risk of functional decline [3, 4], delirium, falls and infections [2] and generates significant costs via emergency room backlogs and delayed surgeries [2, 5]. In addition, individuals may experience reduced quality of life and a financial burden if they are charged a daily co-payment as they wait.

Characteristics of patients with a status of ALC (referred to herein as *patients with ALC*) and risk factors have been studied extensively. Patients with ALC are commonly characterized by cognitive impairment [4, 6–10] including dementia [6, 11], behavioral challenges [12], functional decline [4, 7–9], social vulnerability [13, 14] and advanced age [15–17]. Contextual risk factors for ALC include lack of community services [8, 18] or LTC beds [11, 19–21], lack of timely coordinated care, lack of presence or willingness of family carers [4, 22] and hospital discharge policies which influence length of stay and discharge planning capacity. Few studies have captured the perspective of patients with ALC or their carers [3, 23–26] and no studies could be located that capture the ALC carer experience of patients with cognitive impairment. This perspective is important given that those with cognitive impairment tend to be among the smaller subset of patients with ALC who have the longest lengths of stay with fewest options outside hospital [9] notwithstanding the unique challenges placed on carers.

Patient and carer experience studies generally describe ALC as isolating and uncertain [3, 24, 25] where care markedly drops off [25]. While some patients resign and accept the uncertain process as ‘normal’ others yearn for autonomy and routine and struggle to accept the status quo [21]. In a study by Kydd [25] patients noted that the moods and lack of attention from staff negatively impacted their experience. In a study by Swinkels and Mitchell [3], patients observed that those who did not demand care tended to be favored. In many studies, patients were designated ALC because they were waiting for facility based LTC. While some patients accepted that they were going into LTC, or appeared resigned to it [23] others felt that the decision was made for them [3]. The body of research that incorporated the carer perspective noted that carers continued to provide care while patients were in hospital [23], information about next steps was slow and lacking [24, 26] and, similar to patients, carers felt that the LTC waiting process was uncertain [24].

The purpose of this study was to understand the hospital experience of carers of patients who require an ALC, specifically LTC. We targeted carers of people who lacked decision making capacity or had significant cognitive impairments. Capturing this particular experience is fundamental to improving the quality of care for the most vulnerable and will add insight into a presently small evidence base.

ALC is a designation that is typically devoid of care entitlements, thus important questions arise: 1) *Should care entitlements cease when someone requires an ALC*? And 2) *What is the appropriate balance of responsibilities between medical care staff and informal* (*family*) *carers in meeting the needs of these patients*? We explore these questions in the discussion section of the paper.

## Methods

### Design and setting

This is an exploratory qualitative descriptive study [27] which entails relatively straightforward descriptions of phenomena that are data driven (descriptions stay close to the surface of the data). Consistent with qualitative descriptive methods, a theory or framework was not chosen a priori to guide the data analysis process, nor was a theory generated from the results [28]. Qualitative descriptive methods are appropriate for understudied areas of research; exploratory research as well as those geared to applied health researchers, policy and practitioner audiences. The descriptive content derived from the analysis can be used as a practical guide for both future research and front line quality improvement initiatives in hospital and community settings.

The study took place in Northwestern Ontario (NWO), the largest geographic area within Canada’s most populous province (Ontario) but also the most sparsely populated; home to just 2% of the provincial population while covering 47% of Ontario’s land mass. Approximately 16% of the population in NWO is 65+ and this is expected to increase to 22% in the next ten years [5]. The rapidly aging population is shaped by out-migration of younger adults who are leaving to seek employment in larger urban areas [1, 2]. NWO has the highest proportion of patients with ALC in Ontario [6]; approximately one in four acute hospital beds (26.9%) and one in three post-acute hospital beds. The majority of patients with ALC are waiting for LTC placement in hospitals in Thunder Bay, the largest urban area in the region and the site of this research.

### Participants

Participants had to meet the following inclusion criteria: informal carer of a patient with ALC (i.e., waiting for LTC from a hospital bed); English speaking; could give informed consent; caring for a client of the Province’s publicly funded homecare organization - Community Care Access Centre (CCAC); and caring for a person who was unable to participate in the study due to cognitive decline or limited decision making capacity.

### Recruitment

Our team worked with a North West CCAC staff member to recruit carers. The staff member generated a list of eligible carers and contacted them by telephone using a script provided by the research team. Amenable carers had the option of contacting the researcher directly or having the researcher contact them. All opted to have the researcher contact them. The researcher subsequently contacted the carer by telephone to explain the study, answer questions and if appropriate, set up an interview time. All participants were given a $10 gift certificate to a local coffee shop as a token of appreciation for their time (Table 1).

**Table 1:** Carer Background Information

Variable	*N* = 15^a^ (for 12 unique patients)
Sex	Female = 9
	Male = 6
Relationship to Care Recipient	Spouse = 2
	Child = 7
	Parent = 2
	Sibling = 1
	Relative (non-immediate) = 3
Employment Status	Employed = 7
	Retired = 4
	Not Working = 1
Type of Hospital of Care Recipient	Acute = 1
	Post-Acute = 11
Living Arrangements	Lived with Care Recipient = 3
	Lived Apart from Care Recipient = 9
Length of Hospital Stay of Care Recipient	<1 year = 7
	1–3 years = 4
	>3 years =1
Primary Reason for Hospitalization of Care Recipient	Fall = 5
	Stroke = 1
	Cancer – 2
	Acquired Brain Injury = 1
	Mental Illness = 1
	Frailty (no specific disease noted) = 2
Received Community Services Prior to Hospitalization	Yes = 7
	No = 5 (1 refused services)

^a^In two interviews more than one carer participated

### Data collection

Data collection took place between October 2015 and February 2016. A member of the research team (MM) interviewed 15 carers (across 12 interviews) using a semi-structured interview guide consisting of 13 open ended questions (see Additional file 1). Questions were structured to capture the characteristics of carers (e.g., demographical information, relationship to care recipient, years of caregiving, etc.), perceptions of factors that led to ALC, previous homecare service use and needs, hospital experiences and their experience of the LTC waiting list process. This paper focuses exclusively on the hospital and LTC waiting experience from the carers perspectives. Other parts of the interview guide (e.g., community service needs) will be detailed in a separate paper. The interview guide was developed by the lead author and vetted through local key stakeholders (North West CCAC and hospital staff) to ensure the appropriateness and relevance of questions. The interviews typically ranged between 30 and 60 min in length. All but three interviews took place in person (in the homes of the carers) with the others conducted over the telephone to accommodate out-of-town carers. Interviews were conducted in a one-to-one format with the exception of two interviews that were conducted with multiple carers present (siblings and partners). The interviews were audio-recorded and transcribed verbatim. The period of data collection and analysis overlapped allowing for the assessment of saturation of content. While interviews provided unique examples of experience they fell into consistent thematic areas (detailed below).

### Data analysis

After each interview was completed it was transcribed by an external source and then checked for accuracy by a member of the research team (JI) against the original audio recordings. The transcripts were anonymized (names of individuals and organizations were removed). The interviews were read several times by the lead author and a preliminary codebook was generated. This was achieved by reading the transcripts line by line and making note of units of meaning (codes). A second researcher (JI) reviewed a sub-set of interviews in depth and coded the interviews using the codebook. The researchers met to discuss and refine codes until a consensus codebook was created. This codebook was used to code the full set of interviews. At this stage, the authors used NVivo 10 software to electronically code the interview transcripts. Codes that addressed the hospital experience of carers, including the experience of preparing for LTC were selected for further, in-depth review by two researchers who documented key themes from the text. The researchers met to discuss, refine and reach consensus on a final set of themes. Trustworthiness of data was achieved through prolonged engagement with the data (reading the transcripts multiple times and discussing themes in a series of meetings), and having two reviewers independently review transcripts, generate and reach consensus on themes. There was general alignment in perspectives on the types of content (categories and codes) from the interview data. This discussion was critical to protect against inadvertently excluding or misinterpreting content. The naming of codes was deliberated. Reflexivity (making note of biases and expectations prior to and during coding) was an important piece of the analysis process and characterized discussion amongst the authors, who each have different levels experience with the population of study (research and personal experience) and potentially different expectations of the data. For example, the lead author, based on previous work, expected that carers would resist a long-term care admission and as the results indicate, this appeared not to be the case.

## Results

Five core themes were identified from the interview data: patient over person (the non-medical needs and characteristics of patients’ were ignored); uncertain and confusing process (the length of the waiting time was uncertain and the decisions made by formal care providers did not make sense to carers); inconsistent quality in care delivery (carers were generally frustrated by the lack of time and oversight from care providers); carers addressing the gaps in the system (carers continue to provide care for their loved ones and advocate for their needs); and personalization of long-term care (carers, for the most part, were eager for their loved ones to be placed into long-term care as long as the setting catered to the patients’ needs and preferences and was located close to family). The first three themes captured what carers *observed* when the patient was ALC. The fourth theme captured what carers *did* while the patient was ALC and the final theme captured what carers *wanted* post ALC. Below we describe each of the themes and include illustrative quotes taken from a range of participants. Quotes with […] refers to text removed due to length and redundancy.

### Patient over person

When patients have an ALC designation they are no longer in an acute phase of treatment. Despite the acute phase of treatment being over they continue to have other needs related to personal care (activities of daily living, assistance with meals) and comfort. Carers understood why staff prioritized patients with urgent and acute care needs but felt that their family members should not be ignored. Carers were frustrated when “little things” (such as basic personal hygiene) were not attended to because they were important aspects of personal dignity. It appeared that responding to personal care needs (as opposed to medical intervention) was a priority for carers of patients at this stage.

“*I look at certain times*, *you know*, *where my dad is not tended to*. […] *And I know it*’*s not easy*. … *They*’*re not shaved*, *they*’*re not combed*, *they*’*re not dressed properly*.” Carer 5

Carers noted that patients need encouragement and prompting from care providers in order to have their needs met but these personal touches to care were uncommon.

“…*he won*’*t ring the bell if he has to go to the bathroom or he won*’*t tell you if he has pain*. … *you have to encourage him* – *Do you have to go to the bathroom*? *Or take him every 2 to 3 h. And they told me*, “*Well*…” Carer 6

Carers understood the behaviors of patients, their histories and roles. For instance, a carer was surprised when a case manager did not realize that her mother was a former nurse. The carer felt that her mother (who was not keen about going into long-term care) likely knew what was happening when being assessed and potentially shaped the information she shared with them (presented as more independent than she actually was).

“*Well*, *my mom didn*’*t like her much*. [*laughs*] *And my mother being an old retired nurse*, *she was pretty feisty. And I was a little surprised that this lady didn*’*t know that my mom was a retired nurse and that she had worked at* [*organization name*]. […] *like once we got into the conversation*, *my mom could relate to exactly what was happening*.” Carer 10

Likewise, other carers shared insight on the factors that triggered patient behaviors. A carer, who used to be a hospital nurse, explained why her Mom may have been acting out.

“*I said even if she were to fall*, *break her hip*, *get pneumonia*, *die*… *And that may sound harsh but that*’*s the reality* […] *as long as she had that quality of life where she could still get around and do things*. […] *the reason why she*’*s screaming and all this sort of stuff is because she can*’*t get out of her chair. You guys have buckled her in. She*’*s used to being independent and walking around*. […] *I*’*d much rather see her fall with her walker than be bound in a wheelchair*.” Carer 8

In this case it appeared that protecting a patient from harm (a fall) by buckling her in her seat impacted her autonomy and dignity, resulting in a responsive behavior. These and other dignity and risk trade-offs were observed by the carers and represented the priorities of staff (safety) versus the priority of patients and carers (freedom and dignity).

Some carers felt that the hospital setting was poorly aligned to the perceived needs and preferences of the patients. The hospital environment and pending LTC placement appeared to be particularly difficult for younger patients who resisted living in an environment with “old people.”

Among older patients, particularly those with dementia, the hospital environment was also resisted at times. For instance, the site of one of the ALC wards was a former psychiatric hospital which carried a certain stigma for patients:

“…*because she doesn*’*t want to go back into that place. She thinks it*’*s for the crazy people* […] *And I keep telling her*, *no*, *mom*, […] *it*’*s the overflow* […]. *And she just doesn*’*t*… *She knows but then she doesn*’*t know. You know what*, *within 2 min*, *she says*, “*No*, *no*, *no*.” *But it*’*s hard*.” Carer 12

In summary, carers suggested that the *person* tends to be overlooked within an environment geared to the *patient*. Carers delineated the small but important things that warranted more attention from health staff as well as critical trade-offs that occurred. These trade-offs (particularly between safety and personal independence and dignity) had implications for the quality of life and care experience of patients and carers.

### Uncertain and confusing process

After a period of time in hospital, all patients were placed on the waiting list for LTC. Some carers were unclear if LTC was the best option. For instance, one carer was hesitant about placing his wife into LTC after visiting one of the sites:

“*But after I seen one*, *I said no way*, *I*’*ll look after her. If I have to quit working*, *I said I*’*ll look after her. I can*’*t in good conscious warehouse her in a facility like that and forget about her. No way. We don*’*t do things like that*.” Carer 4

Providers, at times, disagreed as to where the patient should wait for LTC. In one case a carer felt relieved when a specialist sided with her and informed a community care coordinator that the patient could not go home.

In some cases hospital and CCAC staff encouraged or pressured carers to take the client back home as they waited for LTC. In all cases this was met with resistance. A carer recalled a discussion with her son regarding the suggestion from hospital staff that her husband return home to wait for LTC:

“*Yeah. Well*, *they seemed to be very pushy in*, *you know*, *getting him out of there*, *out of the* [*hospital*]. *I guess they needed the beds or something. And he* [*her son*] *says*, “*Mom*, *I think we*’*ve been railroaded*.” *We didn*’*t like the way it was going. They were suggesting that he come home. And we said no because I don*’*t know if I could cope with that and I think it would have been too much for me*.” Carer 1

Being on a waiting list for LTC did not ease feelings of uncertainty. While carers were able to select their LTC home preference and switch their choices thereafter (sometimes with consequences such as being placed further down the list), when and how the placement would actually take place was unclear. One carer feared that a facility would refuse her son given his high care needs. Many were confused and frustrated about the selection process for LTC, particularly how people were prioritized on the waiting list:

“*We know people who have been there less time and been exactly where they wanted to go. And maybe there*’*s more spots for women than men*, *I don*’*t know* […] *No one will tell us anything. You phone* [*community agency*] *and it*’*s all*, “*Oh*, *you know*, *he*’*s on the list*.” *I*’*ve been told that because he*’*s safe and taken care of at* [*hospital*], *he*’*s not a concern to move him* […] *And I feel when the time comes to get everyone out of there*, *he*’*s just going to be stuffed somewhere*.” Carer 6

In one case, a carer started to prepare for her father to come home which entailed making structural adaptations to his home and purchasing equipment. As her father’s needs worsened the discharge destination was switched from home to LTC and it was determined that he should not leave hospital as he waited. Changes in the patient’s health and various inputs from members of the care team created an uncertain and confusing time for this carer as well as some unexpected expenses.

Waiting for LTC was often characterized by multiple moves within and between hospitals. A carer talked about the implications of this for her father with Alzheimer’s disease:

“*But I just didn*’*t think it was fair for my dad having dementia or Alzheimer*’*s to go from*… *You go from one unit and you*’*re settled*, *and you*’*ve got these nurses. Then you go from there*, *you go to 3*-*south*, *to 2*-*south*, *to 2*-*north*, *to 4 and then 5. So he went for 5 moves*.” Carer 6

Patients who had longer periods of ALC were often charged a co-payment as they waited for a LTC bed from the hospital. Issues of fairness were raised among caregivers who had mixed feelings about being charged for their stay despite receiving little to no service.

Ultimately, carers commented that being placed on a wait-list should not follow a “one size fits all” approach:

“*And I think when they*’*re looking at the cases*, *they need to remember that everybody is different* […] *Even though your process in how you get them say into a nursing home is the same*, *you can*’*t say that you do that for everybody. You can*’*t send everybody home and put in services*, *and expect them to be able to stay at home*.” Carer 15

In summary, being put on a waiting list for LTC as well as the location where patients should wait was not always agreed. If and when consensus was reached the process was still unclear. Multiple moves within and between hospitals and feeling “pushed out” were common experiences. The process of being put on a waiting list seemed almost mechanical without much individual tailoring during the process.

### Inconsistent quality in care delivery

Carers noted that the care delivery experience and interactions with staff were of mixed quality. While many had positive interactions with staff, they were overshadowed by frustrating moments. For instance, carers noted that providers did not have time to give the care that they felt their loved ones needed:

“*The staff here are wonderful but they*’*re just over*-*worked. They don*’*t have the time to sit with each person that*’*s on that floor to help them with their meals*.” Carer 2

Some carers believed that lack of care and attention led to poor health outcomes:

*And I find that* [*hospital*] *deconditioned my dad*. […] *not taking him to the bathroom as often*, *and finding him like on the side of the bed with his cast almost down to his knee or past his knee because that*’*s where his injury was*, *because he had to go to the bathroom. And he didn*’*t know better to ring*.” Carer 6

Some carers described how their experiences changed at different points of the patients care trajectory, particularly as they transitioned from having medical needs to having an ALC status. A carer for her son with an acquired brain injury (ABI) was generally satisfied with the care received before he became ALC. As an ABI patient he was surrounded by a multidisciplinary team including mental health providers and social workers who were able to guide the family as they made critical decisions regarding the patients care. The carer observed a change in the quality of care when her son was transferred to the ALC ward:

“*The care is very good. I have found with some transfers*, *we*’*ve sort of had to keep our finger on the pulse of things just to make sure that the continuity of care*… *And I did mention that to one of the ward managers*, *that I found that some things kind of fell through the cracks when there was transfer*…” “Carer 14

Some carers lacked trust in the hospital staff and were skeptical about what happened when they were not around:

“*When she was in* [*hospital*], *I don*’*t know whether they gave her some medication or what but she was so confused. She was talking about something that wasn*’*t even making any sense* […] *But when I talked to them about it*, *they just kept saying she*’*s just mixed up*.” Carer 3

In summary, carers had mixed views about the quality of care received. Carers noted a stark contrast between ‘pre ALC’, which tended to be more coordinated and timely and ALC care when needs were neglected. Some carers lacked trust in the care staff and were uncertain about whether important tasks were completed in their absence.

### Carers addressing the Gaps in the system

Carers continued to provide care while their loved ones were in hospital despite feeling stressed and discouraged. In addition to caring for their family member in hospital, carers had other responsibilities including paid employment, child rearing, caring for other family members and, in a few cases, managing their own disabilities. While the vast majority expressed feelings of frustration, stress and fatigue others appeared more resigned, and perhaps accepting of their circumstances, even if not ideal. In one interview, where 3 children caregivers participated, feelings of guilt were expressed, not as a result of caring for their father who was the care recipient with ALC, but from their mother who continued to live at home, with dementia, and did not agree with the care decisions they made. In all situations carers continued to provide care for their loved one in hospital, particularly by doing the activities that the hospital staff did not do (at least not in the way that the carers felt sufficient). These activities included assisting or encouraging their loved ones with activities of daily living, meals, going for walks and socialization (e.g. visiting, taking them home on a weekend pass, and activities in hospital if available).

A carer stated that she wished the hospital did more “*so it*’*s not all up to the family member*.” Carer 11. This carer associated such lack of activity with decline: “*You might be continent when you arrive but you*’*re incontinent when you leave*.”

Formal providers, at times, expected carers to do tasks that they did not have time to do, including mobility activities:

“*I*’*m thinking*, *well*, *maybe he should get more rehab. Well*, *I had a comment said to me* [*from provider*], “*Well*, *then you know what*, *why don*’*t you come and walk your father*?” Carer 6

This carer expressed a great deal of frustration at the expectations that providers had of the family. She felt that her father was declining functionally due to lack of activation. She coped by advocating for his needs.

Advocating for the needs of patients was common among caregivers, particularly adult children as opposed to aging partners. They effectively ‘worked the system’ to get a desired outcome. In some cases, carers were currently in (or retired from) health care provider roles which afforded them an ‘insider’s view’, helping them navigate the system and ask the right questions. For instance, a carer noted that things improved after questioning providers about the lack of care her Mom received after a fall which occurred while in hospital:

“*And she was just lying in bed for 3 days*, *a little 86 year old. Like no*, *I wasn*’*t very happy* […] *I wasn*’*t angry*, *I wasn*’*t upset. Once I asked the questions*, *I got the best services. The nurses were awesome. Just nobody seemed to know what was happening next*.” Carer 10

This same carer was alerted that a bed had opened up in a LTC facility prior to her Mom’s fall. The carer, who lived across the country, flew to the hospital where her Mom was located and went ahead and moved all of her belongings into LTC without permission so she would not lose her bed.

Other carers described their actions as more complacent and felt that their passivity put them at a disadvantage. When trying to understand how people were prioritized on the waiting list for LTC, a carer felt that it came down to “…*how much noise you make. We*’*ve been pretty calm*.” Carer 7

In summary, the carer role continued while the patient was ALC. Carer activities ranged from meeting personal care needs to advocating for the patient, even from afar, and filling the gaps of care not addressed by the formal care system. Carers who did not speak up felt that they were at a disadvantage.

### Personalization of long-term Care

In the process of waiting for LTC (or following placement in a couple of cases) carers shared what was important to them about this next destination. Many patients were in wards (rooms with multiple beds as an ALC) and frequently changed units and floors to accommodate other patients.

A son caring for his father contrasted the private room in LTC to the hospital ward.

“*He*’*s got his own room. Because at the* [*hospital*], *he was in a 4*-*bed room. So now he*’*s got his own private room*, *and he loves it*.” Carer 13

One carer noted that getting her Mom to a LTC facility and personalizing her space was important to her and to the well-being of her Mom:

“*Yes. I wish we can get her in there fast. It would be better for her. Then I can bring some of her stuff*, *like her TV and her chair that she likes to sit in* […] *At least it makes her look like she*’*s in her own place* […] *It might settle her down more*.” Carer 12

A participant who was caring for his father commented that once his Dad was placed he started to re-engage in things that were previously important to him:

“*Yes*, *there was church options*…*But he just chose not to go. But they never pushed there*, *eh*…*If he didn*’*t want to do it*, *he didn*’*t want to do it* […] *But at* [*Long*-*term Care Facility*], *it*’*s a little different set*-*up here than at the* [*Hospital*]. *I don*’*t know what it is. Just the fact that it is an actual real senior citizen home*, *that might have played on his head too. But he*’*s a little more into participation* […] *And now he*’*s going to church and doing his exercises and things*.” Carer 13

Carers talked about the importance of the patient being in an environment that truly aligned with their needs and preferences. In one case, a carer described her brother with long-term memory impairment as an intellectual person who loved to learn and travel. Having stimulating conversations and books to read were important to him but the LTC home fell short of meeting these needs for her brother.

“[…] *there are so many people that are in wheelchairs*, *they*’*re sleeping. It*’*s just not*… [*Brother*] *says it*’*s not nice to look at people like that. He said*, “*I can*’*t look at people that are like that all day*.” […] *Because he*’*s got a good long term memory*, *he could talk about any philosophy or history that you wanted him to*.” Carer 15

Finally, proximity of the LTC home to the carer’s residence was a factor that was raised to increase the convenience of visits, particularly for those who were working, were raising children or had their own personal health issues. Keeping a spousal unit together was deemed vital by a carer who reflected on the importance of having her mother (who required LTC) close to her father, who also had care needs, close together:

“*I was thinking about*, *well*, *maybe*, *you know*, *if mom goes into* [*LTC home*], *and then wouldn*’*t it be wonderful if dad was in whatever*, [*assisted living closeby*] […] *So that then he wouldn*’*t have to drive and he would have that built*-*in social circle* ….” Carer 11

In summary, when carers reflected on their expectations for LTC, finding an environment that aligned with the needs and preferences of their loved one, having a private space and being in close proximity to family was important for ease of visiting, socializing and patient quality of life.

## Discussion

When patients have a delayed hospital discharge (status of ALC) they have been medically cleared for discharge yet remain in hospital because a more appropriate care setting (such as homecare or long-term care) is not available. ALC is a particularly challenging problem in rural, remote and smaller urban communities (including our study setting) where supports in the community including primary informal carers tend to be in short supply.

Few studies have incorporated the carer perspective and our study is the first of its kind to focus exclusively on the carer experience of patients with ALC and cognitive impairment- a particularly vulnerable sub-set of the ALC population with longer than average lengths of stay [9]. Capturing the experiences and views of carers is critical so they can shed light on the care of their loved ones who may not have the capacity to speak or advocate on behalf of themselves. Similar to previous studies, carers felt that the process of waiting for LTC from the hospital was uncertain and frustrating [7, 26]. Despite there being a systematic process to being wait-listed for LTC, an unclear trajectory ensued. The continued role that carers played while patients were ALC is aligned with findings by McCloskey [19] who found that hospitalization did not provide relief to caring duties. By exclusively focusing on carers our study unpacked detail on what carers do for patients (daily living supports including meeting basic needs) and the tensions that arise when negotiating these roles with formal care providers.

We further discuss our findings in relation to two key questions raised in the introduction of this paper: *1*) *Should care entitlements cease when someone requires an ALC*? And 2) *What is the appropriate balance of responsibilities between medical care staff and informal* (*family*) *carers in meeting the needs of these patients*?

Canada’s publicly funded health insurance model is governed by the Canada Health Act (CHA) which specifies that insured Canadians have access to hospital and physician care based on need and not ability to pay. Publicly funded care outside of hospitals and by non-physicians is at the discretion of each of the provinces who have constitutionally defined authority over the provision of health services for most of their respective populations. The CHA specifies that for people to be eligible for hospital and physician care they must require *medically necessary* care. While medical necessity has not been explicitly defined in the CHA, it has come to be associated with acute, episodic and disease oriented treatment and rehabilitation and not necessarily other things which may enhance health (such as instrumental activities of daily living, ongoing mobility support post rehabilitation, social supports, supports for carers, etc.). Ironically, these ‘non-medical’ supports may *prevent* the need for medically necessary care. When a patient is ALC, despite being within the walls of the hospital, they no longer require medically necessary care. Such a conundrum was recognized by carers who understood why busy staff attended to those with acute and rehabilitative care needs. Nevertheless, carers were frustrated at the lack of attention afforded to their loved ones, which impacted their personal comfort and dignity and in some cases caused further decline. Supports for meals, mobilization and socialization were met haphazardly or not at all. Carers filled these gaps, by assisting with these tasks as well as advocating for their loved ones. Certain carers with a working knowledge of the health system tended to advocate heavily for their family members and at times, got the results they needed. Other carers, who did not have the time, capacity or will to engage in advocacy found themselves at a disadvantage. This finding raises important implications for carer engagement in the health care system. If the ultimate goal of the health system is to improve quality of care then determining ways in which less vocal carers can be meaningfully engaged is crucial to realizing this vision.

Carers noted that lack of attention to non-urgent needs had important quality of care implications. The quality of care implications appeared to materialize in a number of trade-offs such as the prioritization of safety (most important for providers), over choice, freedom and independence (important for carers). Such trade-offs require further attention, as standardized hospital reporting and patient safety may have major implications for person centered care (providing care in line with the goals and preferences of patients and their families).

Such dilemmas beg the question regarding *who should be responsible for what within the walls of the hospital*? Are non-medical support needs the responsibility of formal, paid, hospital providers or the responsibility of informal, unpaid family members, friends and volunteers? Perhaps it is the responsibility of both parties? In our study, carers stepped up to fill these gaps, but at times they did so reluctantly. In some cases, formal providers articulated an expectation that the carers *should* step up to fill these tasks while carers, on the other hand, thought it should be part of the formal hospital provider role. The uncertainty of roles suggests that creating a space to negotiate and manage expectations is warranted, particularly since poor patient and carer outcomes may result. Another point of contention was the co-payment charged to patients for waiting in hospital. This payment was questioned by carers given that patients were not receiving the same level of services that they would in long-term care (where co-payments are also required, but accompanied by a basket of services).

Our study results suggest some potential benefits for examining or even formalizing the role of carers in the health system. Carers have critical insight into the patient that can be used to enhance patient care and assessment of need, particularly for people who cannot speak on behalf of themselves due to significant cognitive impairment or language barriers. Carers were able to articulate why patients behaved in certain ways as well as the types of needs they had, information that simply might not be available to formal care providers who may not have the same history, level of trust established and insights that carers do. While this extends beyond our study findings, there may be times and situations where it is not appropriate to engage family members and other informal care supports in the care of the patient, particularly if such involvement can do harm or put the well-being of the patient at risk. Tools and supports to help formal care providers determine when and how to engage carers may be useful in optimizing patient care and managing carer burnout.

Carers also need to be considered as individuals worthy of care alongside patients. While supports for carers do exist, they tend not to be woven into the fabric of health systems which predominantly cater to the needs of the patient. By the time patients reach the point of being ALC, carers may be at a breaking point. Such stress was evident when carers resisted care providers’ suggestions for patients to return home to wait for long-term care instead of the hospital. Such resistance may have also stemmed from the other roles that carers were filling simultaneously including paid employment, caregiving for others, financially supporting dependents, among other tasks.

Future research may benefit from moving beyond the collection of personal experience to active engagement of carers in the development of strategies to improve care transitions for vulnerable populations as well as supports to enhance their role. Ideally, proactive approaches would be embedded that target patients and carers before they become ALC so delayed hospitalization can be replaced (or prolonged) through ongoing, tailored supports in the community.

### Limitations

While a diverse array of carers (partners, parents, children, extended family, of various ages and living circumstances) across three hospitals participated in this study all were located in one community. Carers were predominantly white and English speaking limiting the transferability of findings to similar geographic settings and carer profiles. Future research, engaging with carers in larger urban areas and across culturally diverse populations and health systems will add greater insight into the carer experience and how it may change according to context.

## Conclusions

Waiting for long-term care from the hospital is a stressful and uncertain time for family caregivers. ALC is an ‘in-between’ phase when patients and carers may be at their most vulnerable yet receive the least care from the formal care system. Carers provide critical insight into the needs and behaviors of patients as well as processes that need to be improved to enhance their experience. Such insights will help health systems internationally as they grapple with the issue of ALC whilst trying to optimize engagement with patients and their families.

## Additional file

Additional file 1: Caregiver Interview Guide. The data collection tool for the study. For this paper, the focus was on the caregiver hospital experience and long-term care planning (thus the latter part of the interview guide was most applicable to the analysis reported in this paper). A separate manuscript will be prepared detailing community care needs and experiences. (DOCX 21 kb)
