# Supplementary material for: “It’s a waiting game” a qualitative study of the experience of carers of patients who require an alternate level of care
Source: BMC Health Serv Res. 2017 May 2;17:318. doi: 10.1186/s12913-017-2272-6 (PMC5414131; doi:10.1186/s12913-017-2272-6)
Supplement: Additional file 1: — Caregiver Interview Guide. The data collection tool for the study. For this paper, the focus was on the caregiver hospital experience and long-term care planning (thus the latter part of the interview guide was most applicable to the analysis reported in this paper). A separate manuscript will be prepared detailing community care needs and experiences. (DOCX 21 kb) [file 12913_2017_2272_MOESM1_ESM.docx]

**Caregiver Interview Guide**

Note: For this paper, the focus was on the caregiver hospital experience and long-term care planning (thus the latter part of the interview guide was most applicable to the analysis reported in this paper). A separate manuscript will be prepared detailing community care needs and experiences.

**Introduction**

1. Tell me a bit about yourself [prompt: are you from the area? How long have you lived in [city]?

**Caregiver Characteristics and Relationship to Care Recipient**

1. What is your relationship to [care recipient] [prompt: spouse, adult child, neighbor, friend]?
2. How long have you been providing care for [care recipient]?
3. Do you live with [care recipient]?
   1. If no, does [care recipient] live alone?
4. What is your relationship with [care recipient] like? [prompt: do you get along most of the time?]

**Health Status**

1. What kinds of health conditions does [care recipient] have?
   1. How long has [he/she] had these health conditions?
   2. Have [his/her] health conditions gotten worse over time?

**Factors that Led to Hospitalization**

1. Tell me about the events leading up to [his/her] most recent hospitalization [prompt: did he/she experience a sudden health event? Who decided that he/she should go to hospital?]

**Community Supports**

1. It is my understanding that [care recipient] was receiving services in the community? If not applicable skip to question 8b.
   1. From your perspective, were these services meeting [his/her] needs? If not, what could have been done differently in order to meet [his/her] needs?
   2. Are there services that [care recipient] needed but could not get? [prompt: could anything have made it easier for you and [care recipient] to manage your health, such as health or social services?] If so, what could have been done differently to meet [his/her] needs?
   3. Did you have a person who works in the health care system, such as a care coordinator who you or [the care recipient] could contact if you had questions?
2. As a caregiver, do you feel that you are/were supported in your role? [prompts: are there services or programs that would make it easier for you to provide care?
   1. Do you (or did you) have to give up/scale back on your own personal activities such as work or hobbies, to provide care?
3. If [care recipient] was transferred back to the community, would you be able to continue on in your role as a caregiver? Explain.

**Long-term Care Planning**

1. Did someone in the community (such as a healthcare worker) talk to [care

recipient] about long-term care (i.e., nursing home care) such as putting [his/her] name on a waiting list before [he/she] came into the hospital? **IF NO, SKIP to question 8**

- 1. If so, were your involved in this discussion too?
  2. How did you and [care recipient] feel about this conversation? [prompt: was the timing right, are there reasons why [care recipient] was not put on the wait-list? Did you and [care recipient] disagree on what should be done? other reasons?]

1. Do you think anything could have been done, along the way, to change the

circumstances that [care recipient] is in today?]

1. Is there anything else that you would like to share with me?

***End of Interview***
